# White Ginseng Ameliorates Depressive Behavior and Increases Hippocampal 5-HT Level in the Stressed Ovariectomized Rats

**DOI:** 10.1155/2019/5705232

**Published:** 2019-02-11

**Authors:** Daehyuk Jang, Hyun-ju Lee, Kyungjun Lee, Kyu-Ri Kim, Ran Won, Seung Eun Lee, Insop Shim

**Affiliations:** ^1^Department of Physiology, College of Medicine, Kyung Hee University, Seoul 02435, Republic of Korea; ^2^Department of Science in Korean Medicine, Graduate School, Kyung Hee University, Seoul 02435, Republic of Korea; ^3^Department of East West Medical Science, Graduate School of East-West Medical Science, Kyung Hee University, Kyunggi-do 17104, Republic of Korea; ^4^Department of Biomedical Laboratory Science, Division of Health Sciences, Dongseo University, Busan 47011, Republic of Korea; ^5^Department of Herbal Crop Research, NIHHS, RDA, Eumsung 27709, Republic of Korea

## Abstract

Postmenopausal depression is closely associated with depletion of estrogen which modulates transmission of 5-HT, a key neurotransmitter that regulates stress-managing circuits in the brain. In this study, antidepressive efficacy of white ginseng (*Panax gingseng Meyer, *WG) was evaluated in stressed ovariectomized rats. Female Sprague Dawley rats were ovariectomized and repeatedly restraint stressed for 2 weeks (2h/day). Thirty minutes before restraint stress, rats were administered saline (control), WG 200 mg/kg (p.o.), WG 400 mg/kg (p.o.), or fluoxetine (PC, 10 mg/kg, i.p.). Tail suspension test (TST) and forced swimming test (FST) were performed to assess antidepressant effect of WG. After behavioral tests, levels of serum corticosterone (CORT) and hippocampal 5-HT were measured. Significant decrease of immobility time in TST and FST was shown in rats administered with PC or WG 400 compared to the control. WG200-treated rats showed remarkable reduction in immobility time of TST. PC, WG 200, or WG 400-administred group exhibited significant reduction of CORT compared to the control. PC or WG-treated rats exhibited remarkable increase in hippocampal 5-HT concentration compared to the control. Hippocampal 5-HT levels in WG groups were higher than those in the PC group. The present study demonstrated that WG had antidepressant efficacy in an animal model of menopausal depression. Treatment with WG enhanced hippocampal 5-HT level while suppressing depressive symptom and serum CORT level. These results provide evidence that WG plays an important role in activating serotonergic neurons in stressful situation, suggesting that WG might be a reliable natural alternative of antidepressant drugs to treat menopausal depression.

## 1. Introduction

Postmenopausal women experience a number of physiological and psychological changes that are closely associated with decreased circulating estrogen due to depletion of ovarian follicle [1, 2]. Since estrogen influences transmission of neurotransmitters involved in the pathophysiology of depression, menopausal women have high risk of developing depression [3]. Particularly, estrogen enhances serotonergic activity by stimulating serotonin (5-hydroxytryptamine, 5-HT) synthesis and transmission and downregulating degradation of 5-HT [4]. Serotonergic neurons from the brainstem innervate to several brain regions including the hippocampus which is well-known to play an important role in regulating cognitive functions and pathological development of mood disorders [5]. A variety of 5-HT receptors and reuptake transporters are expressed in rodent hippocampus. They are involved in maintaining homeostasis of brain function [6]. It has been shown that selective serotonin reuptake inhibitors (SSRIs) that are predominant antidepressants can ameliorate depressive symptoms of patients with major depressive disorder (MDD) by blocking reuptake of 5-HT and stimulating 5-HT receptors in the hippocampus [6]. Decreased hippocampal 5-HT level is accompanied by depressive behavior including sleep disturbance and anhedonia in stressed rats [7, 8]. Enhancement of hippocampal 5-HT activity can reduce depressive-like behavior of stressed and ovariectomized rats [9].

Ginseng (*Panax ginseng Meyer*) contains over 20 ginsenosides (steroidal and nonsteroidal saponins) and nonginsenosides as active compounds [10]. Several studies have demonstrated that ginseng possesses diverse pharmacological activities, including antidepressive, antitumor, antiasthma, neuroprotective, and memory-improving effects [10–13]. White ginseng (*Panax ginseng Meyer*, WG) is a sun-dried and nonfermented ginseng. It has been revealed that ginsenoside contents of WG are Rg_1-2_, Rb_1-3_, and R_c-f_ [14, 15]. WG has been reported to have therapeutic effects on hyperlipidemia, obesity, cancer, and Alzheimer's disease [14–18]. Nevertheless, antidepressant effects of WG in an animal model of menopausal depression have not been reported yet.

Thus, the objective of the present study was to examine antidepressant effect of WG in female ovariectomized rats repeatedly exposed to restraint stress. Depressive behaviors were evaluated through tail suspension test (TST) and forced swimming test (FST). Serum corticosterone (CORT) and hippocampal 5-HT levels were assessed by enzyme-linked immunosorbent assay (ELISA).

## 2. Materials and Methods

### 2.1. Subjects and Stress Procedure

Female Sprague Dawley rats aged 10 weeks (body weight: 180-200 g) were used (Samtako Animal Co., Seoul, Korea). These rats were housed under a 12 h light/dark cycle (lights on at 8 am) with controlled temperature at 22-24°C. They were allowed access to food and water* ad libitum*. All experimental procedures were approved by the Institutional Animal Care and Use Committee of Kyung Hee University (approval number: KHUAP(SE)-13-041) and conducted in accordance with the US National Institutes of Health guidelines (Guide for the care and Use of Laboratory Animals, 8th edition, revised 2011).

Rats were randomly assigned to the following four groups: ovariectomized and restraint stressed with saline-treated group (Control), ovariectomized and restraint stressed with white ginseng (200 mg/kg, p.o.) treated group (WG200), ovariectomized and restraint stressed with white ginseng (400 mg/kg, p.o.) treated group (WG400), and ovariectomized and restraint stressed with fluoxetine (10 mg/kg, i.p.) treated group (PC). Ovariectomy was performed under general anesthesia with pentobarbital sodium (50 mg/kg, i.p.). A small dorsal midline incision was made and bilateral ovaries were dissected under aseptic condition. After incision suture, postoperative recovery was monitored daily for one week. Restraint stress was produced by forcing animals into a cone-shaped PVC to restrict forward, backward, and lateral movements for 2 hr a day for 2 weeks.

### 2.2. Drug Treatment

Rats were administered 200, 400 mg/kg of WG (p.o.), or 10 mg/kg of fluoxetine (i.p.) 30 min prior to stress procedure for 2 weeks. WG powder containing 5.4 mg/kg of R_g1_ and R_b1_ was purchased from Daedong Korea Ginseng Co. (Chungnam, Korea). It has been reported that 5 mg/kg of ginsenoside R_g1_ and 8 mg/kg of R_b1_ have antidepressant effect in a chronic stress-induced depressive animal model [19, 20]. In addition, it has been shown that 100 mg/kg of WG powder containing 10.44 mg/g of ginsenosides possesses neuroprotective effect in memory-deficit rat [14]. Thus, 200 and 400 mg/kg of WG (1.08 and 2.16 mg/kg of ginsenosides, respectively) were chosen in this study to examine their ameliorating effect on depressive-like behavior. Fluoxetine, an antidepressant of the selective serotonin reuptake inhibitor (SSRI) class, was used as a positive control (PC) in this study.

### 2.3. Behavioral Tests

#### 2.3.1. Tail Suspension Test

On the 14th day of drug administration, FST was performed as previously described [21] to assess antidepressant effect of WG. Briefly, rats were placed in a transparent plexiglass cylinder (20 cm in diameter x 50 cm in height) containing water at room temperature to a depth of 30 cm. Immobility time was considered when rat remained floating without movements except to keep its head above the water. It was recorded for 5 min and regarded as depressive-like behavior.

#### 2.3.2. Forced Swimming Test

On the 14th day of the drug administration, FST was performed as previously described [21] to assess antidepressant effect of WG. Briefly, rats were placed in the transparent plexiglas cylinder (20cm in diameter x 50 cm in height) containing water at room temperature to a depth of 30 cm. Immobility time, which considered when the rat remained floating without movements except to keep its head above the water, was recorded for 5 min and regarded as depressive-like behavior

### 2.4. Measurement of Corticosterone (CORT) Level in the Serum

After behavioral tests, animals were anesthetized with sodium pentobarbital (80mg/kg, i.p.). Cardiac blood was collected and centrifuged to separate serum. Obtained samples were stored at -80°C until assay. Levels of CORT in serum samples were analyzed by enzyme-linked immunosorbent assay (ELISA) kit (Enzo life sciences, New York, USA) according to the manufacturer's instructions

### 2.5. Measurement of 5-HT Level in the Hippocampus

After collecting cardiac blood, brains were immediately removed and coronally sectioned using a brain matrix (ASI instruments Inc., MI, U.S.A). The hippocampus (AP, -1.92 to -3.24 mm; ML, ±0.00 to ±4.00 mm from the bregma; DV, -2.40 to -4.20 mm below the dura) was punched out on an ice-cold plate and stored at -80°C until assay. Obtained tissues were homogenized with protein extraction solution (iNtRON Biotechnology, Inc., Gyeonggi-do, Korea), incubated at room temperature for 30 min, and then centrifuged at 10,000×g for 15 min at 4°C. Supernatant was collected to a fresh tube and 5-HT concentration in duplicate aliquots was measured using an ELISA kit (Labor Diagnostika Nord, Nordhorn, Germany) following the manufacturer's instructions.

### 2.6. Statistical Analysis

Statistical comparisons among different groups were analyzed using one-way analysis of variance (ANOVA) followed by Tukey's post hoc test. Data are presented as mean ± standard error of the mean (SEM). All statistical analyses were performed using IBM SPSS Statistics 23.0 software. Statistical significance was considered when* p* values were less than 0.05.

## 3. Results

### 3.1. Effect of WG in the Depressive-Like Behavior

**Figure 1:**
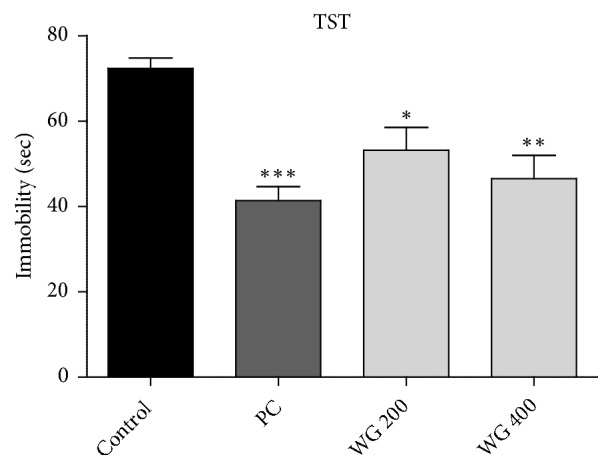
Effect of WG administration on immobility time in the tail suspension test (TST) (n=6 per group). *∗*P<0.05, *∗∗*P<0.01, and *∗∗∗*P<0.001 versus control.

**Figure 2:**
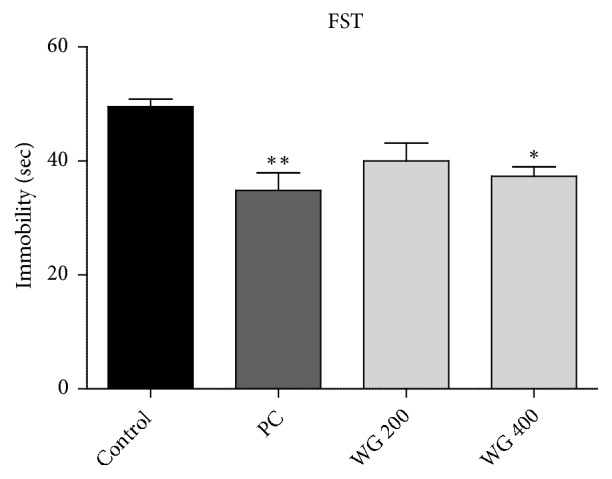
Effect of WG administration on immobility time in the forced swimming test (FST) (n=6 per group). *∗* P<0.05 and *∗∗* P<0.01 versus control.

The immobility time indicating depressive-like behavior was significantly different among groups in both TST (F_3,20_ = 9.714,* p *< 0.001, Figure 1) and FST (F_3,20_ = 6.987,* p* < 0.01, Figure 2). Fluoxetine as a positive control (PC) significantly reduced immobility time in TST and FST compared to the control (*p* < 0.001 and* p* < 0.01, respectively). Rats administered with WG 200 and WG 400 also showed significant decrease of immobility time in TST (*p* < 0.05 and* p* < 0.01, respectively, Figure 1). In FST, WG 400-treated rats showed remarkable reduction in immobility time compared to the control (F_3,20_= 9.714,* p* < 0.05, Figure 2) whereas WG 200-treated group only exhibited a tendency to decrease. These results indicate that WG can alleviate depressive behavior in a dose-dependent manner.

### 3.2. Effect of WG in the Serum CORT Level

**Figure 3:**
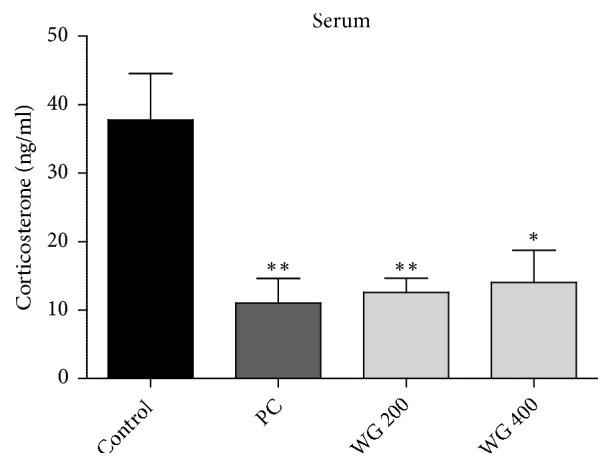
Effect of WG administration on corticosterone (CORT) level in serum after restraint stress for 14 consecutive days (n=6 per group). *∗* P<0.05 versus control and *∗∗* P<0.01 versus control.

Serum CORT concentrations in control and drug-treated rats were analyzed. CORT levels in control, PC, WG 200, and WG 400 groups were 37.79±6.75, 11.01±3.60, 12.56±3.09, and 14.02±4.74 ng/ml, respectively (F_3.17_ = 7.886,* p* < 0.01). As shown in Figure 3, PC, WG 200, and WG 400-administred groups exhibited significant reduction in CORT level compared to the control group (*p* < 0.01,* p* < 0.01, and* p* < 0.05, respectively).

### 3.3. Effect of WG in the Hippocampal 5-HT Level

**Figure 4:**
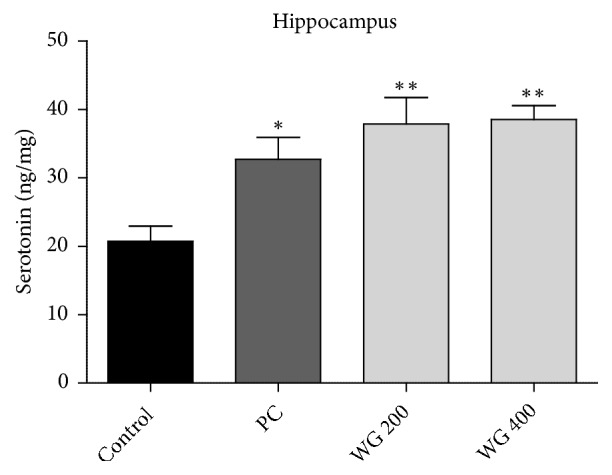
Effect of WG administration on serotonin (5-HT) level in the hippocampus after restraint stress for 14 consecutive days (n=6 per group). *∗* P<0.05 versus control and *∗∗* P<0.01 versus control.

Results of concentrations of 5-HT in the hippocampus region of the brain of control and drug-administered groups are shown in Figure 4. Hippocampal 5-HT levels in control, PC, WG200, and WG400 groups were 20.74±2.23, 32.74±3.21, 37.89±3.86, and 38.54±2.03 ng/mg, respectively (F_3,20_=7.926,* p*<0.01). PC- and WG-treated rats exhibited significantly higher hippocampal 5-HT concentration compared to the control (*p* < 0.05 and* p* < 0.01, respectively).

## 4. Discussion

Depression is an affective disorder with symptoms of psychological, neuroendocrinological, and physiological dysfunction. Many studies have been conducted to find effective antidepressive agents. The central monoaminergic system is a well-known pharmaceutical target of antidepressants. SSRIs are typically prescribed medications to treat major depressive disorder and anxiety disorder. However, they have adverse effects such as nausea, akathisia, sexual dysfunction, and sleep disturbance [22, 23]. The present study demonstrated that administration of white ginseng (WG) could ameliorate depressive like behavior, inhibit increment of stress hormone, and enhance hippocampal serotonergic activity in an animal model of postmenopausal depression caused by ovariectomy and repeated restraint stress. This is the first study to report the antidepressant efficacy of white ginseng. Results of this study suggest that WG is a natural alternative medication to alleviate symptoms of menopausal depression by enhancing serotonergic transmission in the hippocampus and downregulating CORT secretion.

TST and FST are useful and reliable behavioral tests for evaluating depressive symptoms in rodents [11, 24–26]. Decrement in duration of immobility indicates an antidepressant effect of treated-drugs. Fluoxetine, one of SSRIs, was used as positive control (PC) in this study. PC-treated group exhibited significant decrease of immobility time in TST and FST compared to the control, consistent with previous finding [27]. Higher dose (400 mg/kg) WG-administered rats showed much lower immobility time than lower dose (200mg/kg) WG-treated rats in both TST and FST, suggesting that WG could alleviate depressive-like behavior in a dose-dependent manner. These findings suggest that WG is a suitable alternative of SSRIs for reducing behavioral symptoms of depression.

Menopausal transition increases vulnerability to stress and increases the risk of developing mood disorders [28]. Stress responses are regulated by hypothalamic-pituitary-adrenal- (HPA-) axis. Sex-hormones can influence HPA-axis activity. Thus, stress responses of male and female are different. CORT levels in female rats are much higher than those in male rats after stress [29–31]. Increment of CORT level, a consequence of impaired regulation of HPA-axis and hippocampus, is associated with the development of depression [32, 33]. HPA-axis of postmenopausal women is dysregulated due to depletion of female sex hormone, estrogen. Repeated stress causes remarkable alterations in CORT level and depressive behavior in ovariectomized rats compared to nonovariectomized group [34]. Stress-induced increment in CORT level of a menopausal animal model was significantly decreased by treatment with PC and WG in the present study, indicating that WG could act on the hippocampal 5-HT system and ameliorate depressive-like behaviors which in turn might diminish CORT release.

The hippocampus is one of the important brain regions. It maintains mood balance by interacting with HPA-axis. Patients with MDD show hyperactivity of HPA-axis due to impairment of hippocampal glucocorticoid receptor-mediated negative feedback [33, 35]. Chronic stress induced-inhibition of hippocampal neurogenesis upregulates HPA-axis activity [36, 37]. 5-HT is a predominant neurotransmitter that regulates normal hippocampal neurogenesis [38]. Thus, 5-HT acts as an antidepressant by supervising stress-managing circuits in the brain. SSRIs can increase hippocampal 5-HT transmission, resulting in inhibition of depressive symptoms [39]. Our results demonstrate that WG has an antidepressant effect through enhancing 5-HT concentrations. These effects are likely to be mediated by ingredients of WG such as ginsenosides Rb1 and Rg2. For example, Rb1, one of the major active components in WG, has been reported to be able to enhance serotonergic system by increasing 5-HT synthesis, decreasing 5-HT degradation, and stimulating 5-HT_2A_ receptor in the brain [20, 40, 41]. Activity of inhibitory 5-HT_3A_ receptor is suppressed by treatment with Rg2 [42]. Release of dopamine and norepinephrine is also increased by administration of Rb1 in the mouse brain [20]. Rb1, a potential phytoestrogen, exhibits antidepressant effect via increasing 5-HT activity. This effect is mediated by activation of estrogen receptor [40, 43]. Thus, ginsenosides in WG might affect the hippocampal 5-HT system directly through 5-HT receptors and indirectly through estrogen receptor. In the present study, hippocampal 5-HT levels in WG-administrated rats were remarkably increased compared to those in the control, suggesting that WG could alleviate stress-induced depressive behavior by stimulating the serotonergic system in the hippocampus.

## 5. Conclusions

In conclusion, results of this study demonstrate that white ginseng has an antidepressant efficacy in an animal model of menopausal depression. WG can enhance hippocampal 5-HT activity and suppress depressive symptom. Further studies are needed to investigate pharmacological and molecular mechanisms underlying the antidepressant effect of WG on 5-HT biosynthesis, its receptors, and interactions with other neurotransmitter systems. The present study provides evidence that WG plays an important role in activating serotonergic neurons under stressful situation. It also suggests that WG might be a reliable natural alternative of antidepressant drugs to treat menopausal depression.

## Data Availability

The data used to support the findings of this study are available from the corresponding author upon request.
